# A nanometric cushion for enhancing scratch and wear resistance of hard films

**DOI:** 10.3762/bjnano.5.114

**Published:** 2014-07-10

**Authors:** Katya Gotlib-Vainshtein, Olga Girshevitz, Chaim N Sukenik, David Barlam, Sidney R Cohen

**Affiliations:** 1 Department of Chemistry and Institute of Nanotechnology & Advanced Materials, Bar Ilan University, Ramat-Gan 52900, Israel; 2 Department of Mechanical Engineering, Ben Gurion University, Beer Sheva, Israel; 3 Department of Chemical Research Support, Weizmann Institute of Science, Rehovot 76100, Israel

**Keywords:** finite element analysis, hard coating, scanning probe microscopy, scratch, thin films, tribology

## Abstract

Scratch resistance and friction are core properties which define the tribological characteristics of materials. Attempts to optimize these quantities at solid surfaces are the subject of intense technological interest. The capability to modulate these surface properties while preserving both the bulk properties of the materials and a well-defined, constant chemical composition of the surface is particularly attractive. We report herein the use of a soft, flexible underlayer to control the scratch resistance of oxide surfaces. Titania films of several nm thickness are coated onto substrates of silicon, kapton, polycarbonate, and polydimethylsiloxane (PDMS). The scratch resistance measured by scanning force microscopy is found to be substrate dependent, diminishing in the order PDMS, kapton/polycarbonate, Si/SiO_2_. Furthermore, when PDMS is applied as an intermediate layer between a harder substrate and titania, marked improvement in the scratch resistance is achieved. This is shown by quantitative wear tests for silicon or kapton, by coating these substrates with PDMS which is subsequently capped by a titania layer, resulting in enhanced scratch/wear resistance. The physical basis of this effect is explored by means of Finite Element Analysis, and we suggest a model for friction reduction based on the "cushioning effect” of a soft intermediate layer.

## Introduction

Polymers are versatile materials within an extraordinary range of properties. In many tribological applications, they are often preferred relative to metal alternatives [1–3]. However, their relatively low hardness values results in susceptibility to surface damage. Transparent polymers, for example, are widely used in ophthalmology and the automobile industry, but scratches degrade their optical and mechanical properties [4–5].

There are several ways to improve the scratch resistance of polymers. One is to decrease *E*/σ_y_, where *E* is Young's modulus and σ_y_ the yield stress. Materials with a low *E*/σ_y_ ratio are less easily scratched [6]. Another way is to increase the elastic relaxation in elastic–plastic strain by increasing the strain-hardening coefficient [6–8]. Polymeric composites also give substantial improvement in the wear and friction behavior [2].

A third approach, which has the major advantage of not changing the bulk polymer properties is to use inorganic coatings [9–10]. One such attempt to reduce sensitivity to scratching involves depositing an oxide coating on the polymer surface [11]. This solution is challenging due to issues of adhesion between layers and because of the mismatch in elastic strain between the coating and the substrate. A second approach used polysiloxane and acrylic coatings. They have similar elastic strain as the substrate and do provide some scratch protection due to their hardness [6].

**Figure 1:**
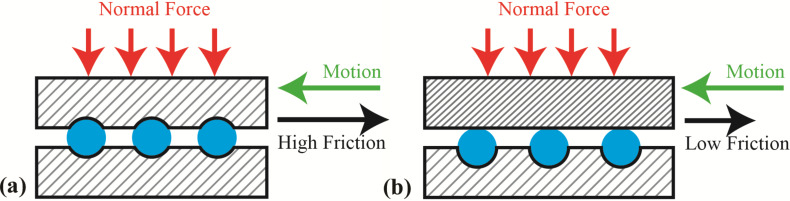
Friction from plowing of wear particles [16] a) materials of same hardness, b) top material harder than lower. Blue balls represent wear particles trapped between the interfaces.

There are literature reports of the effect of hard-on-soft coatings wherein the soft component is only marginally softer and more compliant than the stiffer and harder film [12]. The softer underlying substrate is proposed to promote sliding and energy dissipation during deformation of the hard film. In their classic model of friction, Bowden and Tabor [13] divide the friction into two terms, a plowing term and an adhesion term. The latter is associated with friction arising from the energy required to break the adhesive bonds, and the former from the displacement of plastically-deformed material when a harder surface pushes into and “plows” through the softer one. Plowing leads to release of wear particles due to abrasion of engineering surfaces, which themselves contribute significantly to friction [14]. Figure 1 pictorially demonstrates the phenomenon of friction reduction when one of the opposing surfaces is much harder than the other, as well as the role of wear particles. In order to reduce wear, hard coatings such as metal oxides and hard carbon or diamond coatings are often employed. The adhesion term arises from the growth of junctions forming between asperities on the opposing surfaces, which is influenced by local stress. For hard surfaces, where asperities can reach high stress before breaking or plastically deforming, both of these terms may come into play. Simplistically, one may expect that by providing a cushioning affect, to reduce local stress, both junction growth and plowing could be reduced. Hard on soft/flexible structures also result in a larger threshold for plasticity [15].

Friction has both theoretical and practical interest in materials science in general, and polymer science in particular, with applications ranging from the tire industry [17], to medical catheters [18–20]. Surface modification to control friction is the subject of several patents [21–24].

Many techniques have been applied to study scratch resistance and friction [25–28]. Atomic force microscopy (AFM) allows for extending such studies to the nanoscale while providing high-resolution imaging of the damage caused by wear or scratching [29–32]. The quantitative mechanical measurements require calibration both of the normal and lateral forces. For scratch resistance, only the normal force calibration is necessary. According to the ASTM standard G171 (03) – standard test method for scratch hardness of materials using a diamond stylus [33], scratch hardness changes inversely with the scratch width at a given load.

In the work reported herein, a two-layer coating is used to alter the hardness and friction of a titania overlayer based on the elasticity of an underlying PDMS elastomer. To demonstrate the generality of the strategy, different substrates were coated with titania, with and without a soft underlayer. We have previously reported the deposition of thin and uniform titania films on Si, kapton, and polydimethylsiloxane (PDMS) using liquid phase deposition [34–36]. In this work, we further apply the technique to polycarbonate (PC) substrates, which are used in the lens industry and carry requirement of scratch/wear resistance. In addition to scratch and wear resistance, we demonstrate that the soft polymer underlayer reduces friction. Our study of these effects also extends to examining their variation as a function of the thicknesses of both the hard (titania) and soft (PDMS) layers. We demonstrate a new approach to improving scratch resistance and friction characteristics of many different materials, without modifying their bulk properties. Finite element analysis (FEA) provides insights into the mechanism of friction reduction. Finally, we show the value of AFM-based methods for measuring nanoscale mechanical properties of thin hard films – a system that is otherwise difficult to characterize [37].

## Results and Discussion

**Figure 2:**
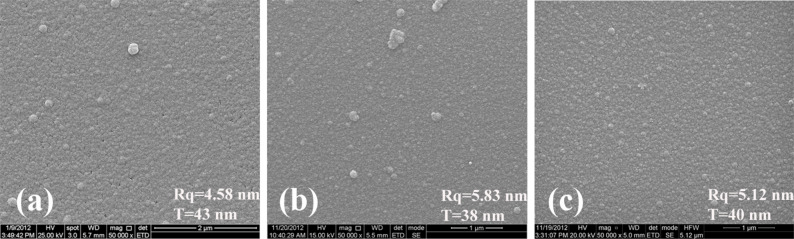
SEM images of titania coating on a) Si, b) kapton and c) PDMS. rms roughness measured by AFM and thickness by RBS.

The initial development of our experimental approach was done using plasma treated Si wafers, kapton and PDMS which were coated with a uniform TiO_2_ nanoscale overlayer. Figure 2 shows SEM images of those coatings. RBS measurements gave TiO_2_ thicknesses of 43, 38, and 40 nm respectively for the various substrates.

**Figure 3:**
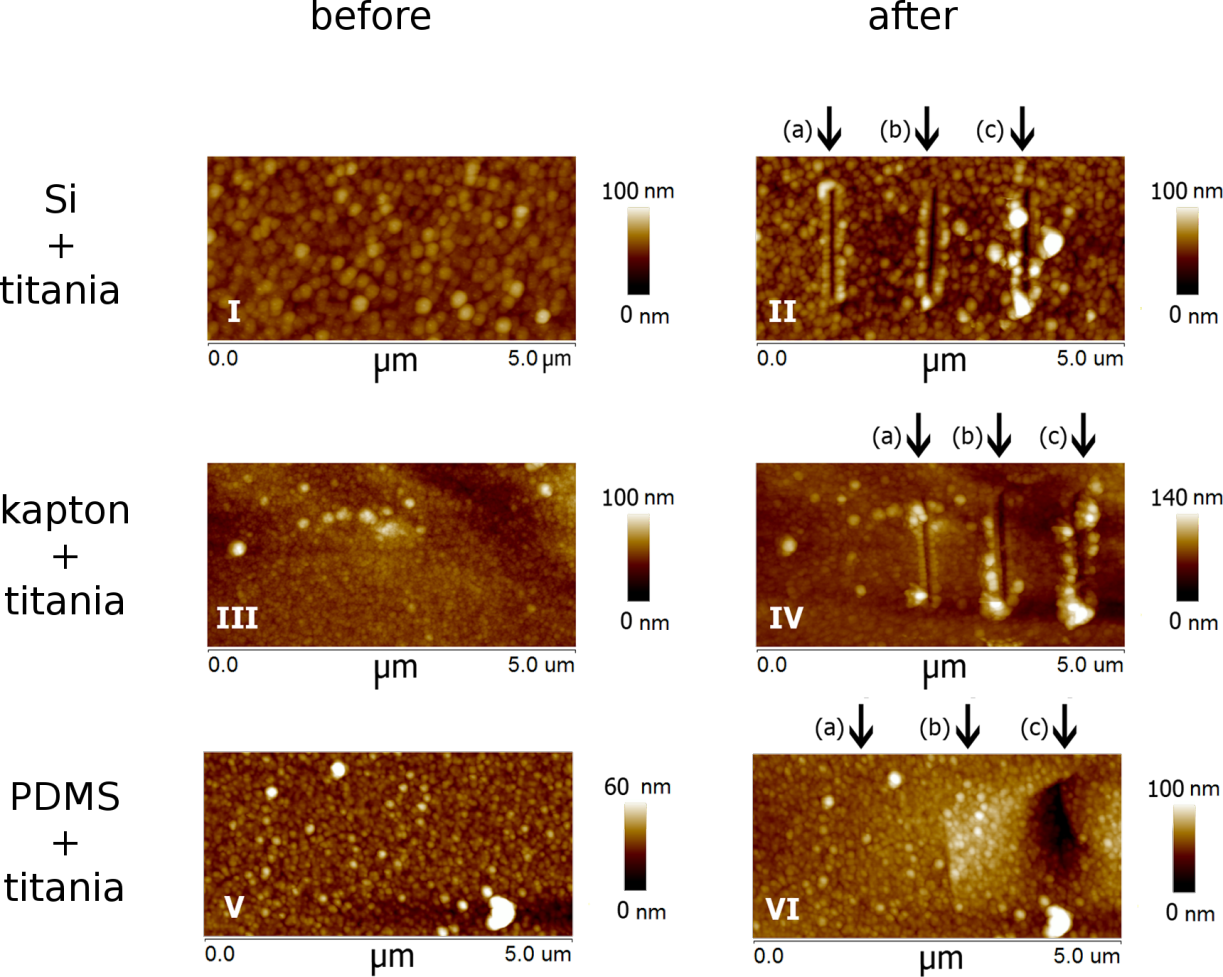
AFM images of titania on Si (I, II), kapton (III, IV) and PDMS (V, VI) before and after scratching loads for the 3 scratch lines were a) 15 µN, b) 20 µN, c) 25 µN.

**Figure 4:**
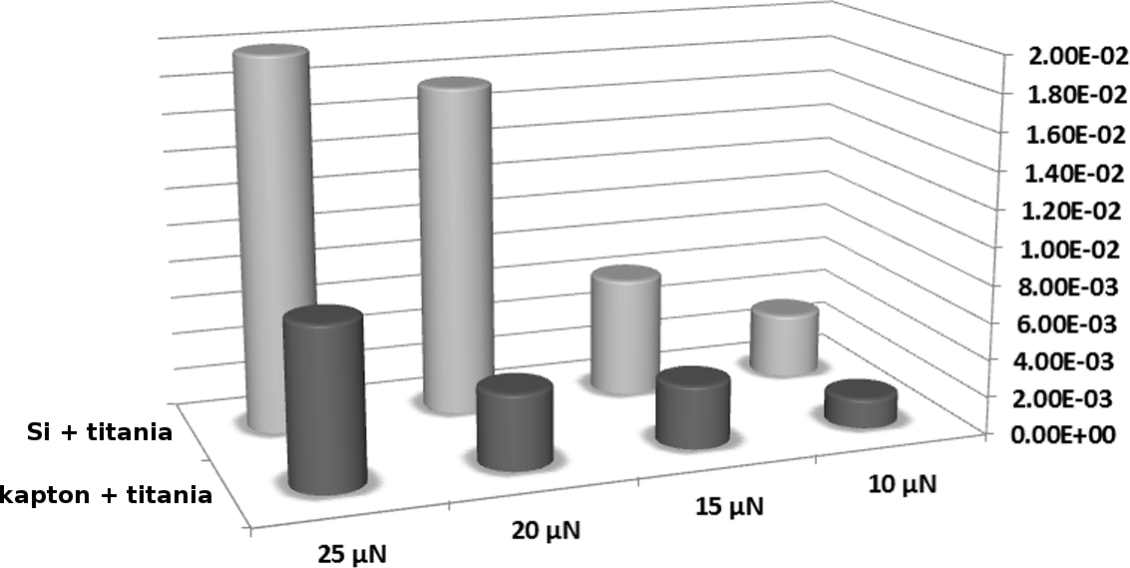
Wear volumes (in µm^3^) resulting from different loads for the titania coatings on Si and kapton.

Scratch tests are routinely used to measure the adhesion of thin film coatings [38]. Figure 3 depicts AFM images of TiO_2_ films on Si/kapton/PDMS before and after scratching. The scratch rate was 0.3 µm/sec and the load was varied from 10–25 µN. It can be seen that all films are disrupted at the maximum load of 25 µN. Titania films deposited directly on kapton and Si wafers were least resistant to scratching. It is clearly seen that titania on PDMS is more scratch resistant than on the other substrates. Furthermore, the volumes measured in the wear test show that titania on kapton performs better than the titania on the Si substrate (Figure 4). These results suggest the beneficial influence of a softer substrate in improving the scratch resistance of the titania films. We refer to this as a “cushioning effect”. Taking the Young's modulus as a measure of the substrate compliance, this quantity varies from 170 GPa for Si [39], to 2.5 GPa for kapton [40] to 20 MPa for PDMS which has undergone plasma activation [37].

**Figure 5:**
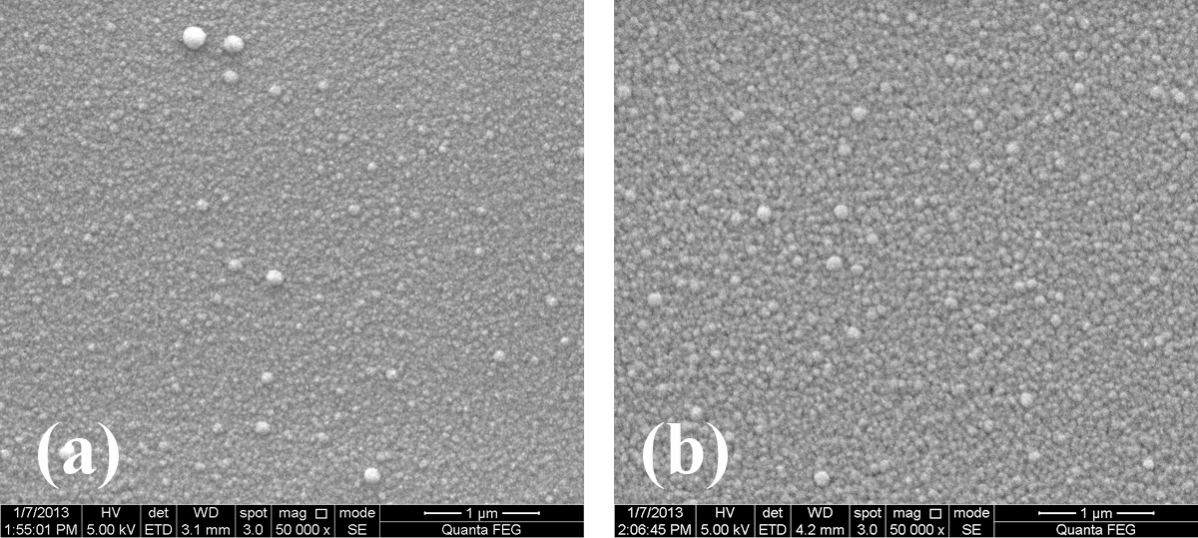
SEM images of titania coating on a) PC, b) PC and PDMS.

**Figure 6:**
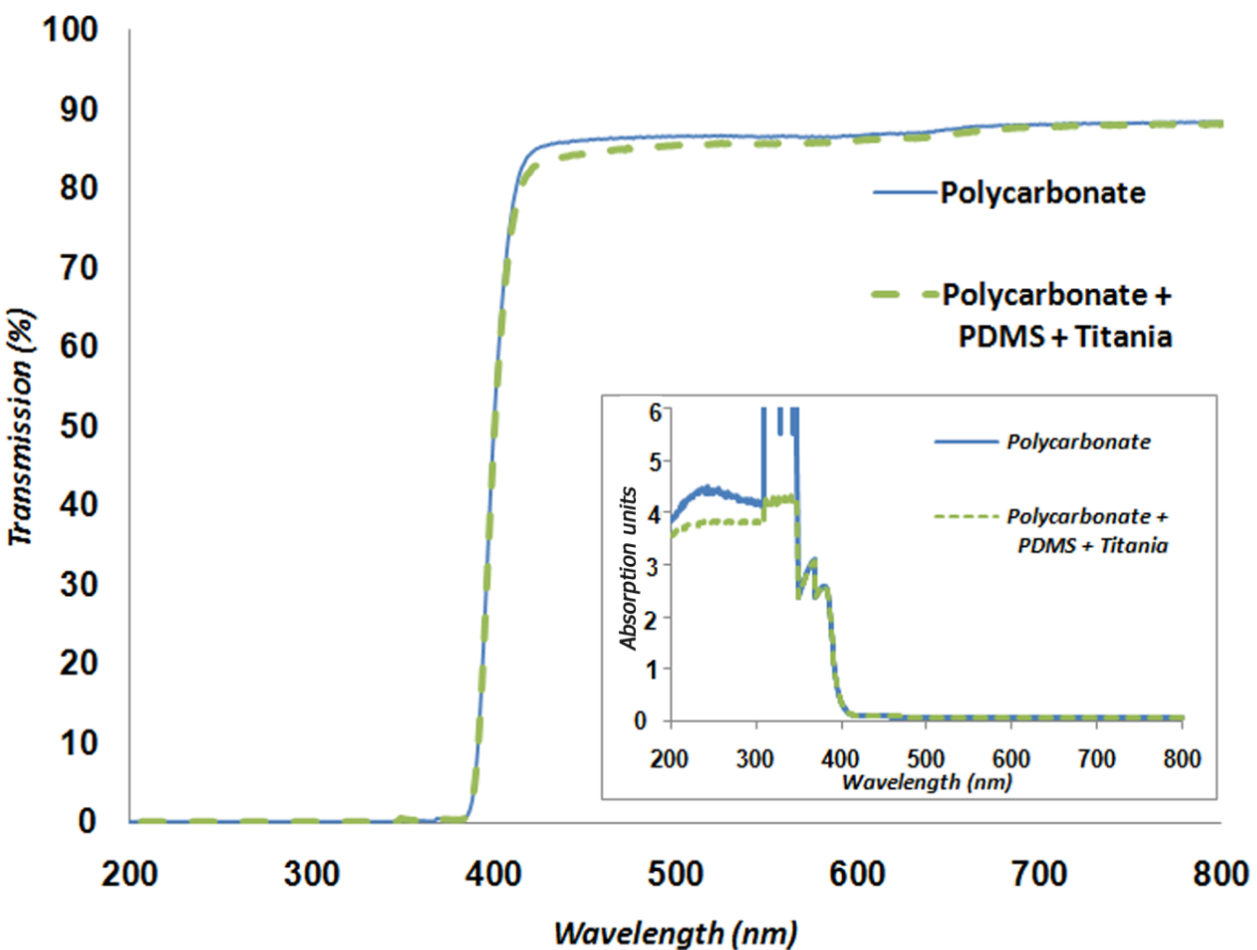
UV–vis spectra for PC with and without titania/PDMS coating. Blue is uncoated PC and green is coated PC; both show no evident absorption in the visible region.

Scratch resistance of titania-coated polycarbonate substrates (with and without a PDMS inter-layer) was measured for its potential relevance to the lens industry. For maximal optical throughput, the thickness of the soft polymer (PDMS) must be minimized. Thinner layers of PDMS were obtained by diluting it with hexane in a ratio of 1:10 followed by spincoating under standard conditions. The hexane evaporated during the curing process. The average thickness of the PDMS layer used for these purposes was about 700 nm, based on cross-sectional FIB measurements. The Young's modulus of PDMS after hexane evaporation was measured with Peak-Force QNM^®^ and found to be the same as in those samples with 10 µm thickness that had been obtained with no dilution with hexane – 1.5 MPa (before oxidative activation). Figure 5 shows SEM micrographs of the titania films on PC with and without the intervening PDMS layer, and Figure 6 the UV–vis spectra for coated and uncoated PC. The PDMS/titania/PC sample has high transmission throughout the visible spectrum, as is the case for uncoated PC which absorbs in the UV [41], but has only negligible absorbance in the visible, as required for good lens quality*.*

**Figure 7:**
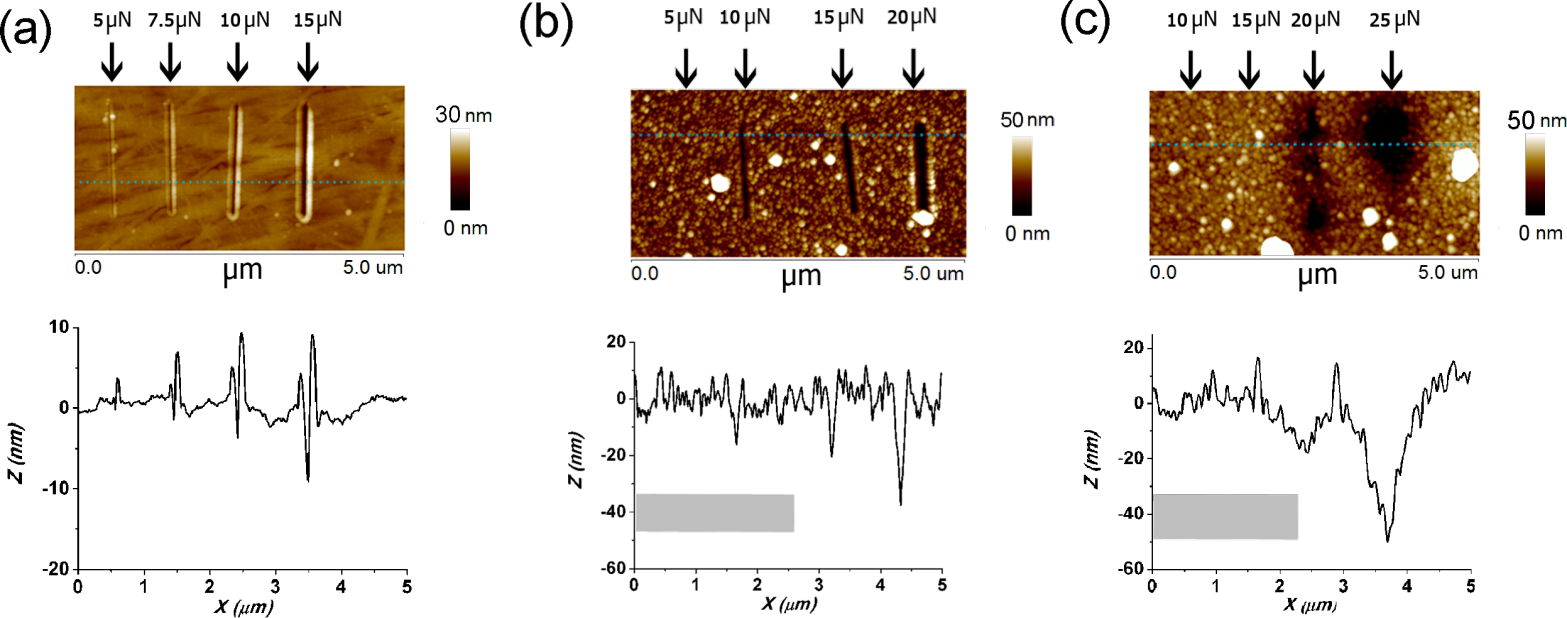
AFM images showing results of scratch tests on coated and uncoated PC at the loads indicated. (a) no coating (b) 40 nm titania coating (c) 40 nm titania on activated PDMS. Cross profiles taken at the dashed lines show the breadth and depth of scratch, with unperturbed surface set to 0 height and scratch depths appearing as negative values. The grey band centered at −40 nm in (b) and (c) shows the approximate PC substrate position and average titania roughness.

Figure 7 shows the AFM images of the scratch tests on PC with and without coatings. The load was varied from 5–25 µN and the scratch rate was 0.3 µm/s. It can be seen that uncoated PC is readily scratched by a 5 µN load. PC with titania can be scratched by a 10 µN load and PC with PDMS and titania is only scratched by a 20 µN load; i.e., the scratch resistance of PC/PDMS/TiO_2_ is twice that of TiO_2_ films on PC, and four times that of the PC substrates.

The scratch profiles in the presence of the PDMS interlayer are qualitatively different than for the bare PC and for titania on PC: for the latter two, the profile is sharp, and defined by the shape of the tip. In the presence of PDMS, wear is inhibited at the lower loads but when a wear track appears at the higher loads it is significantly broader. This can be understood from the width of the deformation predicted by the FEA studies and discussed below. For all scratch tracks except the 25 µN load on titania + PDMS on PC the measured depth was less than the thickness of the titania coating. These results are qualitatively similar to those observed on kapton (Figure 3) which is in line with the similarity in Young’s modulus of kapton and PC: 2.5 and 2.6 GPa, respectively.

**Figure 8:**
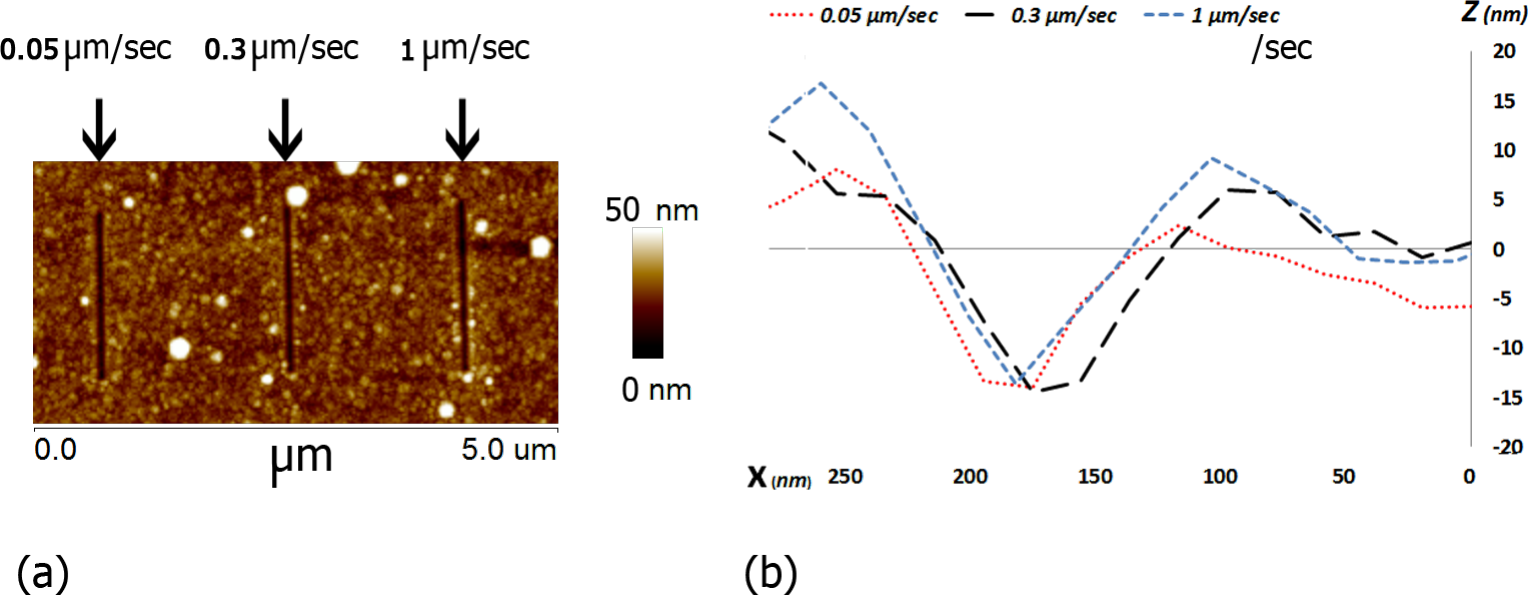
Dependence of scratch resistance on sliding speed. a) AFM image of PC + TiO_2_ after scratching. b) Cross-sections of the scratches at three different velocities and constant load of 10 µN.

The dependence of sliding velocity on scratch resistance was checked for TiO_2_ on PC samples over the range of 0.05–1 µm/s, using a constant normal force of 10 µN. Figure 8 shows that there is no dependence of the scratch depth on the scratch rate in the measured range.

**Figure 9:**
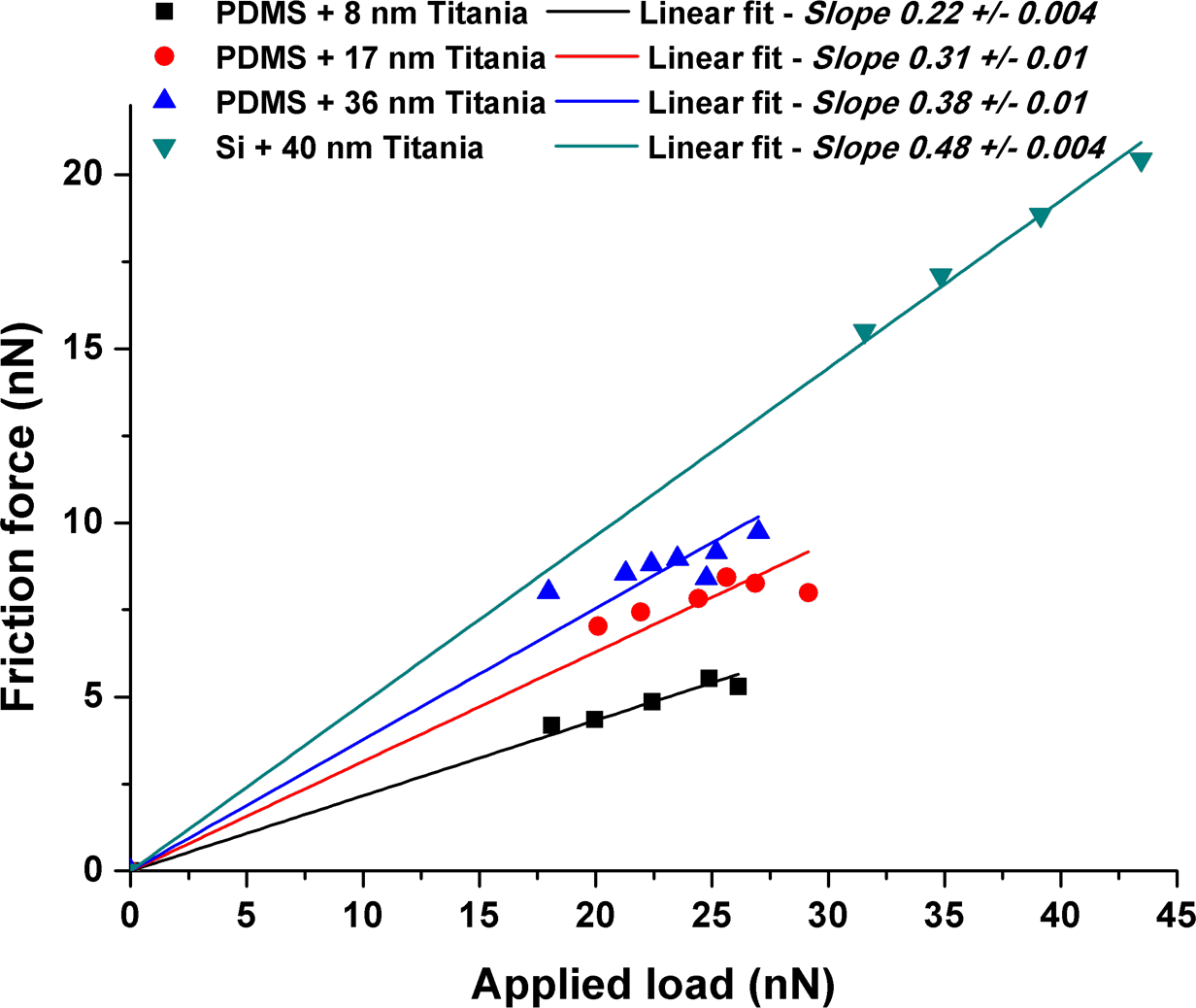
Applied load vs friction force curves and µ evaluated by lateral force microscopy.

Whereas loads in the scratch tests were chosen to scratch away the titania coating, nanofriction measurements were performed at loads that were sufficiently low such that no evidence of wear is observed. This was done so as to avoid plowing and debris generation. The friction coefficient µ is evaluated by


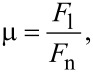


where *F*_n_ is the normal force and *F*_l_ the corresponding lateral force. Following both normal and lateral force calibration, absolute values for the friction coefficient were determined from slopes of lines in Figure 9. In these experiments, the normal contact force was varied in a controlled fashion by changing the feedback setpoint and recording corresponding changes in frictional (torsion) forces. Adhesion as determined from force–distance curves was negligible so the normal force is equal to the applied force, determined from setpoint and cantilever calibration. The normal forces in the friction measurements were 8–30 nN. The presence of a PDMS underlayer is seen to provide a significant reduction in friction coefficient relative to a titania layer alone. Furthermore, µ varies systematically with the thickness of the titania overlayer.

For the thinnest titania coating, 8 nm, µ was 0.21, half that of a plain titania surface. Increasing the titania thickness resulted in a corresponding increase in µ: 0.31 for 17 nm titania and 0.38 for 36 nm titania. The effect is not related to changes in surface roughness [42], as we have previously shown that roughness does not vary with thickness for these films [37]. Nonetheless, local roughness can significantly affect the absolute values of friction coefficient measured in single and multiple asperity friction experiments [43].

Many elastomers are known to exhibit high friction. This is due to the adhesion component of friction, which leads to extensive growth of the junctions between the interfaces under sliding. For instance, the frictional stress in rubber is almost entirely due to adhesive interactions which cause the rubber to fill in the cavities caused by roughness on the mating surfaces. However, the titania coating prevents this adhesion from occurring. The higher yield stress and modulus of the titania relative to the PDMS underlayer mean that higher pressures are required to deform the asperities. These results show that the inclusion of a PDMS underlayer reduces friction relative to a simple titania coating. This effect diminishes as the thickness of the titania increases. The more flexible PDMS layer leads to significant lowering of local stiffness [37]. Thus, the asperities can be deflected downwards without being deformed at their apex which would lead to junction growth. These combined effects of hard film and compliant underlayer can be directly related to the ratio of hardness to elastic modulus. This quotient has proven to be an excellent indicator of wear resistance where larger values indicate better wear resistance [44]. We note that the yield pressure, which is proportional to *R*^2^[*H*^3^/*E*^2^] where *R* is the contact radius, *H* hardness and *E* reduced modulus, has an even stronger dependence on the hardness to modulus ratio. This ratio is enhanced for the titania on PDMS film since the effective *E* is significantly reduced relative to pure titania, as discussed in [37], whereas *H* remains high as dictated by the properties of the titania film. The negligible effect on *H* is due to the fact that the contact area of the indenter in the titania film itself at a given load is not increased here (relative to that for pure titania). The reduced compliance arising from the presence of the PDMS substrate is thus a key factor in wear reduction. Furthermore, the PDMS is below its glassy transition so there is little internal friction. Under this model, the polymer serves as a cushion, which reduces the local pressure at the contacting asperities of the titania, and thus reduces plowing and wear [45]. As the titania layer thickness increases, the surface stiffness increases and the effect becomes less efficient.

**Modeling**. In order to understand the contribution of each component to the total wear behavior the experiment was modeled by FEA. The two-component system was modelled by upper layer and substrate which are assigned the physical properties of the respective materials. Because of the strong bond between film and substrate in the real sample, the two components could be considered glued to one another. This assumption prevents a rupture of the film as is eventually seen for the real physical system, and additionally leads to unrealistically high stress at the interface, but gives the closest approximation to the physical system. The effect of interfacial stress/strain can be observed in each of the modeling runs, and is included in the force balance.

**Figure 10:**
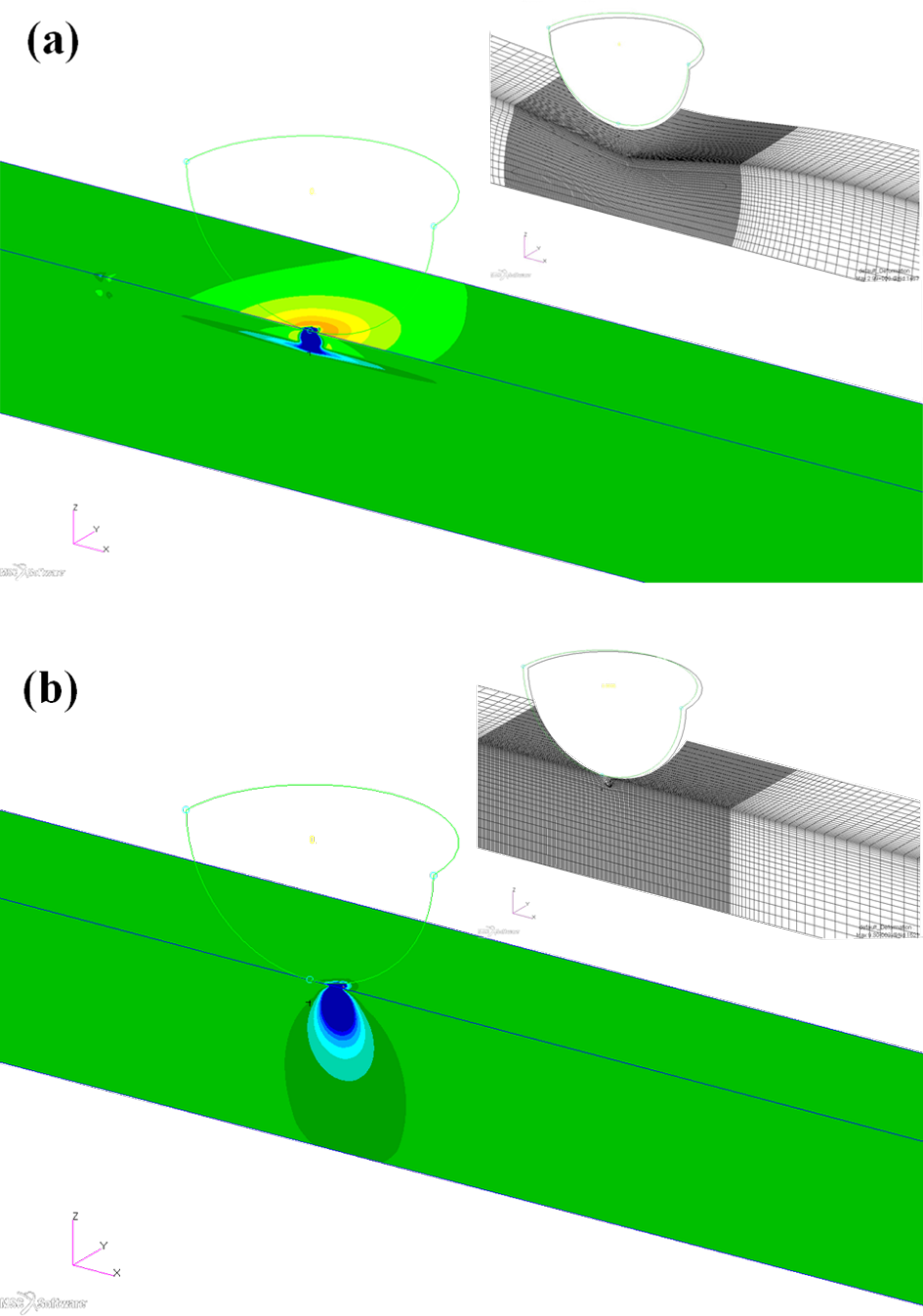
FEA model for 7 nm titania layer for 10 nN force showing *z*-component of global stress distribution for a) PDMS substrate, and b) Si substrate. Inset shows the deformation, with *z* enlarged by 7× for PDMS and 70× for Si. The color scale ranges from 50 MPa tensile (red) to 50 MPa compressive (blue).

Figure 10 depicts the deformation and stress profiles resulting from the FEA model. These mappings give a good insight as to how the substrate compliance influences the behavior. With the stiffer silicon substrate the deformation is localized and small (Figure 10b, inset), whereas on PDMS, the deformation is much more extensive, due to the large deformation within the PDMS substrate (Figure 10a, inset). These differences are reflected in the stress profiles. For a silicon substrate the lack of deformation results in very little tensile stress in the titania film. However, there is significantly larger and more spatially extensive compressive stress within the substrate (Figure 10b). This leads to delamination of the film. For the compliant PDMS substrate, the extensive deformation results in larger tensile stress within the film, and at the film–substrate interface, but much smaller and diffuse stress profile within the substrate (Figure 10a).

The total friction work at onset of sliding, taken as 2 nm of X motion, was also calculated. This value is shown in Table 1. Here, we see clearly that the friction energy dissipated during sliding is reduced to 53% of that for the control Si substrate for the 8 nm titania on PDMS sample, and to 78% of the control for 36 nm of titania on PDMS. This compares favorably to the experimental results of Figure 10, where the friction coefficient for the 8 nm titania on PDMS is 46% that of the control, and for the 36 nm layer 79% of the titania on Si.

**Table 1:** Total FEA friction work at onset of sliding (2 nm X motion).

Substrate/thickness	7 nm	36 nm

Si	3.0·10^−18^ J	3.6·10^−18^ J
PDMS	1.6·10^−18^ J	2.8·10^−18^ J

The robustness of a thin film depends both on its intrinsic strength and the differential stresses that arise between the film and underlying substrate. During standard scratch tests, the film may undergo cracking caused by cohesive failure from tensile stress or spallation due to compressive stress. Thus, a large mismatch between the lateral stresses on the two components will lead to wear and to energy dissipation. The FEA modeling confirms the observed behavior. The modeling points to a mechanistic interpretation: (1) The extensive deformation of the film ultimately allows the stress to relax into a large volume in the substrate, preventing local damage of the surface. (2) The stress directly under the tip at the interface of sliding is larger for the case of silicon substrate. (3) This results in larger work of friction.

## Conclusion

We have shown that scratch and wear resistance of hard films can be improved by introducing a "cushioning" layer of soft polymer. The combined mechanical properties of the polymer underlayer and the hard oxide outer coating provide significant improvement of film wear characteristics. The good adhesion between overlayer and polymer resulting from the liquid phase deposition procedure ensures the robustness of the film. These effects can be tuned by varying titania thickness while preserving the bulk properties and surface chemical properties. AFM/LFM measurements are powerful tools for determining scratch resistance and friction of the hard thin film on the soft polymeric substrate. The FEA model allows us to understand the contribution of each component to the tribological behavior. This understanding can improve the design of composite materials for optimization of wear and friction. This work, in combination with our previous report of variable stiffness for oxides on polymers [37] demonstrates that nanometric oxide thin films deposited from aqueous solution are a powerful tool for controlling the mechanical characteristics of polymer interfaces.

## Experimental

### Titania coating

Coatings were prepared on 1) silicon wafers (n-type) which were cleaned successively by chloroform, acetone, and then ethanol, drying the surfaces in a dry nitrogen stream after each rinse. 2) 125 μm thick kapton 500 HN sheets (DuPont). Prior to titania coating, the as-received sheets were cut into 1 cm × 1 cm squares, washed with double distillated water and ethanol, and dried under nitrogen. 3) PDMS: a mixture of SYLGARD silicon elastomer 184 and SYLGARD silicon elastomer 184 curing agent (ratio 10:1 w:w) was spin-coated onto n-type silicon wafers after cleaning as above, using a Model KW-4A spin-coater (ChemSols Corp.). 4) 1 mm thick polycarbonate PC3107 sheets (Palram). Prior to titania coating, the as-received sheets were cut into 1 cm × 1 cm squares, washed with isopropanol, and dried under nitrogen. Before the titania coating, the surface was pretreated by exposing to an air plasma (Harrick, model PDC-3XG) at a pressure of 0.3 mm Hg and 18 W power for 20 min for Si, kapton and PC and 5 min for PDMS. Immediately after plasma activation, samples were placed in a room temperature titania deposition solution (0.3 M H_3_BO_3_ and 0.1 M (NH_2_)_2_TiF_6_ in water [46]) that was first aged for 6 h and then filtered through a 0.45 µm, 7 bar max filter (Schlecher & Schuele). The deposition was allowed to proceed for 4 h for the 40 nm films and proportionately shorter for thinner films [37]. The aged/filtered solution gave thinner and smoother coatings as compared to coatings done with freshly prepared solutions [36–37]. The TiO_2_-coated samples were rinsed in water and methanol before drying under conditions of controlled humidity [47]. The stability of the coating was confirmed by sonication in water for 10 min.

#### Surface Characterization

**Scanning electron microscopy (SEM).** The surface morphology of the samples was assessed by SEM (Inspect, FEI), at an accelerating voltage of 30 kV with surface gold coatings of approximately 10 nm thickness.

**Rutherford backscattering spectrometry (RBS).** The thicknesses of the TiO_2_ layers were measured by Rutherford backscattering spectrometry (RBS). This work was done using a 1.7 MV Pelletron accelerator (NEC, USA). All spectra were collected using a 2.023 MeV ^4^He^+^ ± 1 keV beam. The beam current was ≈13 nA, with a nominal beam diameter of 2 mm. An electron suppressor was used between the beam entrance and the sample holder, biased at −100V vs ground.

In these measurements the data were collected by the fixed Silicon-Charge Particle Detector (ULTRA^TM^, ORTEC) with detector scattering angle of 2.7 msr. A normal incidentbeam was used in all measurements. All samples were mounted on the holder by double sided, self-adhesive carbon tape. Charging effect on the kapton was compensated by a thin Au coating (8 nm). NDFv9.4e software was used to fit the data [48].

**Focused Ion Beam (FIB).** The PDMS thickness was obtained using a dual beam FIB (FEI, Helios 600), with electron and ion beams operating simultaneously and independently at energies up to 30 kV (52 degrees between the beams) enables simultaneous work using both beams.

**Atomic force microscopy (AFM).** All scanning probe microscopy was done using an ICON instrument (Bruker AXS SAS). The deflection sensitivity of each probe was measured by pressing the probe on a hard surface and spring constant was calibrated by the "Sader method" [49]. Nanoscratching was done using a diamond coated tip (DDESP) (force constant of 20–80 N/m, Digital Instruments, Santa Barbara, CA). Nanoscratching was done with the indenter at variable normal loads of 5–25 μN, a sliding speed of 0.3 μm/s and a scratch length of ≈1.5 μm. The same indenter was used to image the area after the nanomechanical tests. The modulus of the PDMS was measured using PeakForce QNM (Quantitative Nanomechanical property mapping), an extension of the Peak Force Tapping^®^ mode, using ScanAsyst-air probes (force constant of 0.4 N/m, Digital Instruments, Santa Barbara, CA). To quantify the mechanical properties of the sample, the tip radius was determined by measurements on a reference sample of known modulus [50].

The Dimension ICON SPM is capable of measuring frictional forces on the surfaces of samples using lateral force microscopy (LFM). Frictional information was obtained via the torsional deflection of the cantilever with the scan direction running perpendicular to the major axis of the cantilever. Quantitative values of the frictional force were made as per reference [51]. A silicon nitride "A" shaped cantilever (normal spring constant 0.32 N/m) with a gold reflective coating was used for the LFM mode. Nanoscope analysis software was used for analyses of the data.

**Figure 11:**
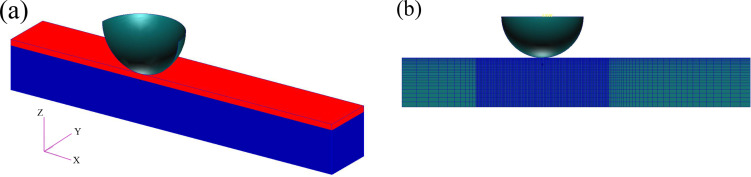
Model used for finite element calculations, for 7 nm titania film thickness. (a) Substrate is seen in blue and titania film in red (b) Mesh distribution of the elements.

**Finite Element Analysis (FEA).** The FEA model chosen to model the experiment used a stiff upper layer of varying thickness, representing TiO_2_, glued to a substrate, representing the PDMS or Si. The model is shown in Figure 11. The substrate width is approximately 440 nm and the mesh is much finer in the vicinity of the indenter than at more distant regions as seen in Figure 11b. The FE model used eight-node hexahedral elements, 58520/128480 and 64240/41800 in the layer/substrate for 7 and 36 nm film thicknesses, respectively. Because of the symmetry, a half model was used with the proper symmetric conditions. Two different thicknesses of the stiff upper layer were modeled 7, and 36 nm. The indenter was a non-deformable spherical tip with radius of 10 nm to conform to SEM images of the tip used for the experiments. The simulation was executed by MSC.MARC FE code, and used a bilinear shear, Coulomb model for the friction.

Contact conditions are defined only between the indenter tip and the TiO_2_ layer. During the simulation, the indenter is first pressed against the layer until the working load is reached, then the lateral force is ramped up. In general, a stick–slip behavior is observed whereby initially there is only insipient sliding until eventually the static friction is exceeded and larger movement occurs. Both substrate and layer are assumed to be 3D isotropic. The mechanical properties used are displayed in Table 2. The friction coefficient between indenter and titania is set as 0.3.

**Table 2:** Young’s modulus *E* and Poisson ratio 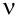 used in FEA calculations.

	Si	PDMS	titania

*E*	170 GPa	20 MPa	65 GPa
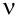	0.22	0.4	0.28

The choice of overall dimensions of the model and size of mesh were chosen for efficient use of computer resources. As our interest was in investigating the static friction, only the initial sliding was studied, up to 2 nm of displacement in sliding direction (X).

## References

[1] Zhang S W (1998). Tribol Int.

[2] Czichos H, Klaffke D, Santner E, Woydt M (1995). Wear.

[3] Shalwan A, Yousif B F (2013). Mater Des.

[4] Briscoe B J, Evans P D, Pelillo E, Sinha S K (1996). Wear.

[5] Jacobson S, Hogmark S (2009). Wear.

[6] Gauthier C, Durier A-L, Fond C, Schirrer R (2006). Tribol Int.

[7] Bucaille J L, Felder E, Hochstetter G (2004). J Tribol.

[8] Bucaille J L, Gauthier C, Felder E, Schirrer R (2006). Wear.

[9] Yaghoubi H, Taghavinia N, Alamdari E K (2010). Surf Coat Technol.

[10] Gilberts J, Tinnemans A H A, Hogerheide M P, Koster T P M (1998). J Sol-Gel Sci Technol.

[11] De Sanctis O, Gómez L, Pellegri N, Parodi C, Marajofsky A, Durán A (1990). J Non-Cryst Solids.

[12] Lackner J M, Major L, Kot M (2011). Bull Pol Acad Sci: Tech Sci.

[13] Bowden F P, Tabor D (1939). Proc R Soc London, Ser A.

[14] Komvopoulos K, Saka N, Suh N P (1987). J Tribol.

[15] Tsui T Y, Pharr G M, Oliver W C, Bhatia C S, White R L, Anders S, Anders A, Brown I G (1995). Mater Res Soc Symp Proc.

[16] Moran J, Sweetland M, Suh N P (2004). Low friction and wear on non-lubricated connector contact surfaces. Electrical Contacts, 2004. Proceedings of the 50th IEEE Holm Conference on Electrical Contacts and the 22nd International Conference on Electrical Contacts.

[17] Heinrich G, Klüppel M (2008). Wear.

[18] Brostow W, Deborde J-L, Jaklewicz M, Olszynski P (2003). J Mater Educ.

[19] Wilkins D P (2008). Catheter tubular components formed of polymer with particles or fibers reducing the friction coefficient. U.S. Pat. Appl..

[20] Lentz D C, Drewes D A (2011). Reinforced catheter or sheath with reduced friction surface. U.S. Pat. Appl..

[21] Bonnizzio D J (2013). Mixture and method for increasing friction coefficient of a surface. U.S. Pat. Appl..

[22] Cox C V, Shaw M C, Epshteyn Y (2012). Low-friction surface coatings containing molybdenum and molybdenum disulfide for metal surfaces. U.S. Pat. Appl..

[23] Zhmud B (2012). Method for providing a low-friction surface of cast iron liner. WO Pat. Appl..

[24] Miikki N, Heiskanen I (2010). Method of treating the surface of plastic-coated disposable container. WO Pat. Appl..

[25] Rudermann Y, Iost A, Bigerelle M (2011). Tribol Int.

[26] Sander T, Tremmel S, Wartzack S (2011). Surf Coat Technol.

[27] Beegan D, Chowdhury S, Laugier M T (2007). Surf Coat Technol.

[28] van Breemen L C A, Govaert L E, Meijer H E H (2012). Wear.

[29] Bennewitz R (2005). Mater Today.

[30] Bhushan B (2005). Wear.

[31] Martinez-Martinez D, Kolodziejczyk L, Sánchez-López J C, Fernández A (2009). Surf Sci.

[32] Yan Y D, Dong S, Sun T (2005). Ultramicroscopy.

[33] Available from: http://www.astm.org/DATABASE.CART/HISTORICAL/G171-03.htm

[34] Pizem H, Gershevitz O, Goffer Y, Frimer A A, Sukenik C N, Sampathkumaran U, Milhet X, McIlwain A, De Guire M R, Meador M A B (2005). Chem Mater.

[35] Gouzman I, Girshevitz O, Grossman E, Eliaz N, Sukenik C N (2010). ACS Appl Mater Interfaces.

[36] Girshevitz O, Nitzan Y, Sukenik C N (2008). Chem Mater.

[37] Gotlib-Vainshtein K, Girshevitz O, Sukenik C N, Barlam D, Kalfon-Cohen E, Cohen S R (2013). J Phys Chem C.

[38] Smolik J, Zdunek K, Larisch B (1999). Vacuum.

[39] Boyd E J, Uttamchandani D (2012). J Microelectromech Syst.

[40] Available from: http://www2.dupont.com/Kapton/en_US/index.html

[41] Moehrle M, Soballa M, Korn M (2003). Photodermatol, Photoimmunol Photomed.

[42] Raman V, Tang W T, Jen D, Reith T R (1991). J Appl Phys.

[43] Bhushan B (2005). Micro/Nanotribology and Materials Characterizaiton Studies Using Scanning Probe Microscopy in Nanotribology and Nanomechanics, An Introduction.

[44] Leyland A, Matthews A (2000). Wear.

[45] Persson B N J (1998). Sliding Friction Physical Principles and Applications.

[46] Deki S, Aoi Y, Miyake Y, Gotoh A, Kajinami A (1996). Mater Res Bull.

[47] Razgon A, Sukenik C N (2005). J Mater Res.

[48] Barradas N P, Jeynes C (2008). Nucl Instrum Methods Phys Res, Sect B.

[49] Sader J E, Chon J W M, Mulvaney P (1999). Rev Sci Instrum.

[50] Available from: http://www.veeco.com/pdfs/appnotes/quantitative-mechanical-property-mapping-at-the-nanoscale-with-peakforce-qnm-an128-lores.pdf

[51] Varenberg M, Etsion I, Halperin G (2003). Rev Sci Instrum.

